# Compressive sampling based on frequency saliency for remote sensing imaging

**DOI:** 10.1038/s41598-017-06834-4

**Published:** 2017-07-26

**Authors:** Jin Li, Zilong Liu, Fengdeng Liu

**Affiliations:** 1 Department of Precision Instrument, Tsinghua University, Beijing, 100084 China; 2 Department of Engineering, University of Cambridge, 9 JJ Thomson Avenue, Cambridge, CB3 0FA UK; 3 Optical Division, National Institute of Metrology, Beijing, 100029 China

## Abstract

In saliency-based compressive sampling (CS) for remote sensing image signals, the saliency information of images is used to allocate more sensing resources to salient regions than to non-salient regions. However, the pulsed cosine transform method can generate large errors in the calculation of saliency information because it uses only the signs of the coefficients of the discrete cosine transform for low-resolution images. In addition, the reconstructed images can exhibit blocking effects because blocks are used as the processing units in CS. In this work, we propose a post-transform frequency saliency CS method that utilizes transformed post-wavelet coefficients to calculate the frequency saliency information of images in the post-wavelet domain. Specifically, the wavelet coefficients are treated as the pixels of a block-wise megapixel sensor. Experiments indicate that the proposed method yields better-quality images and outperforms conventional saliency-based methods in three aspects: peak signal-to-noise ratio, mean structural similarity index, and visual information fidelity.

## Introduction

Remote sensing integrated camera (RSIC) technologies have become a new focus of research in small- and microsatellite photography^1–3^. Compressive sampling (CS) is considered the holy grail of RSIC technologies because it can improve the degree of integration of camera systems, compression systems, high-capacity storage systems, and high-speed image transmission systems. Efficiently performing CS is a key task in RSIC technology. Many CS imaging systems with various characteristics have recently been proposed^4–6^. CS is a novel sampling theory that functions at a much lower sampling rate than the Nyquist frequency^7, 8^. To reconstruct signals from significantly limited samples, CS relies on the fact that most natural signals are sparse and compressible, consistent with a proper basis function. A number of theoretical contributions to CS have been achieved in the past few years. For example, in 2010, Ying *et al*. proposed a saliency-based CS (S-CS) scheme based on the features of the human visual system (HVS) in which sensing resources are allocated to different blocks based on a saliency map^9^. This scheme allocates more sensing resources to salient regions than to non-salient regions to improve the reconstruction quality. S-CS is suitable for remote sensing imaging because remote sensing image interpretation mainly relies on the manual operations of interpreters. To some extent, the perceptual quality of remote sensing images can be influenced by visual attention, and the HVS pays more attention to salient regions of an image than to the rest of the image^10^.

S-CS can be modelled using a cross-correlation sampling system in which the optical signal is simultaneously sampled by a low-resolution (LR) sensor and a high-resolution (HR) sensor. In this paper, a block-wise megapixel sensor (BMPS) is used as the HR sensor. The BMPS is composed of multiple imaging units, each of which is a 16 × 16 pixel image block. Meanwhile, the LR sensor captures LR images. First, the saliency information is detected from the LR sensor images using the pulsed cosine transform (PCT) method. Next, the saliency information is calculated as follows:

1 $${S}_{I}=G\otimes {(abs({C}^{-1}({sign}(C(X)))))}^{2},$$

where *G* represents a two-dimensional Gaussian low-pass filter, ⊗ denotes the convolution operation, *abs*(·) represents the absolute value function, *C* ^−1^ is a two-dimensional inverse discrete cosine transform (DCT), *sign*(·) is the signum function, *C*(·) is a two-dimensional DCT, and *X* is the LR image. On the basis of the saliency information map, the number of sensing measurements can be calculated by applying the saliency monitoring algorithm to each corresponding image block, and then the BMPS processing is performed to complete the sensing sampling process. However, this remote sensing imaging process is subject to several inevitable drawbacks. First, the PCT method can generate significant errors in the calculation of the saliency information because it uses only the signs of the DCT coefficients. Second, the reconstructed images can exhibit blocking effects because image blocks are used as the processing units during the BMPS processing. Third, block-based S-CS in the image domain lacks robustness because it is incompatible with progressive coding, which is an important part of remote sensing applications. Finally, this method depends on an auxiliary LR sensor to perform compressive imaging, and the auxiliary LR sensor and its subsequent processing introduce a calculation process that can increase the load of the camera system.

Based on all of the above considerations, we thus propose a post-transform frequency saliency CS (PTFS-CS) method. In PTFS-CS, a post-wavelet transform to calculate the saliency information, using an approach that is proposed for the first time in this paper, and an optimized S-CS process is added to the calculation to obtain a better-quality image. The post-wavelet transform is defined as a secondary transform in the wavelet domain; i.e., the blocks of wavelet coefficients are further transformed using an orthonormal basis dictionary. The BMPS is composed of an image sensing layer (ISL), a wavelet transform layer (WTL), and a compressed sensing layer (CSL). The ISL acquires the original digital image through photoelectric conversion and analogue-to-digital conversion. The WTL applies a wavelet transform to the original image. The resulting images in the wavelet sub-bands are defined as the BMPS images. The CSL performs saliency-based CS in the post-wavelet domain. First, each pixel of a BMPS image is treated as a wavelet coefficient in PTFS-CS. Second, the spatial frequency in the post-wavelet domain is used to calculate the saliency information. Finally, S-CS is performed in the post-wavelet domain. The proposed PTFS-CS method has the advantages of high accuracy of the saliency information and high imaging quality without blocking effects. This method has potential applications in microsatellites, integrated cameras, and star trackers and also has potential to become a remote sensing CS imaging standard in the future.

## Results

**Figure 1:**
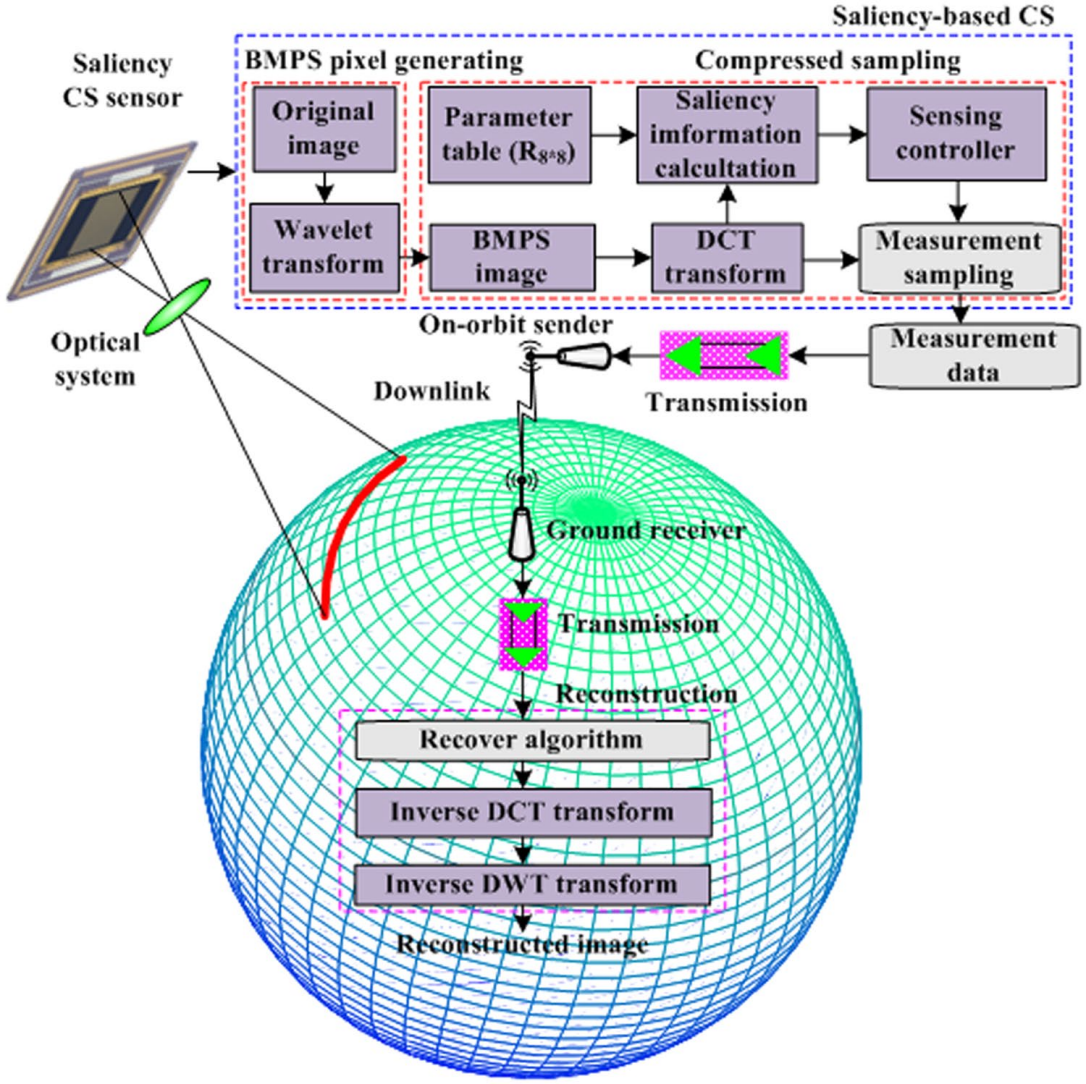
Overall architecture of the proposed PTFS-CS scheme.

The overall architecture of the proposed PTFS-CS method is described diagrammatically in Fig. 1. The PTFS-CS procedure consists of two main parts, namely, CS and ground reconstruction. In CS, a complementary metal-oxide-semiconductor (CMOS) sensor is used to obtain the original image layer, as shown in Fig. 1. A three-level wavelet transform is performed in the original image layer to obtain the BMPS image. Next, a DCT is applied to the BMPS image to obtain the transformed post-wavelet coefficients. Afterwards, the saliency information calculation module obtains the saliency map by using (10). The constant matrices *Q*_4 × 4_ and *R*_4 × 4_, the latter of which is defined in (11), are saved in a parameter table for the saliency calculation. The sensing controller then directs the measurement sampling module to perform sensing sampling on the basis of the saliency map. The measurement data obtained through sensing sampling are transmitted to a ground reconstruction model (GRM) via multiple high-speed optical fibre transmission channels (OFTCs), in-orbit satellite downlink providers, ground receivers, and OFTCs. The GRM reconstructs the DCT coefficients, wavelet coefficients, and original images using a recovery algorithm based on orthogonal matching pursuit (OMP), an inverse DCT, and an inverse discrete wavelet transform (DWT), respectively. Considering the properties of the HVS, the PTFS-CS method exploits saliency information based on spatial frequency in the post-wavelet domain to allocate more sensing resources to salient regions than to non-salient regions. Based on the theory of CS, a recovery algorithm will obtain higher image quality for an image block with more sensing sources; thus, for a given number of sensing measurements for the entire image, the PTFS-CS method can reconstruct the salient regions much better than conventional compressive sampling. In addition, the proposed method is easy to implement. In particular, the saliency algorithm and the sensing measurement sampling process require only multiplication and addition operations, without iterative search algorithms. Such calculations are easy to implement on the hardware platforms of remote sensing cameras.

**Figure 2:**
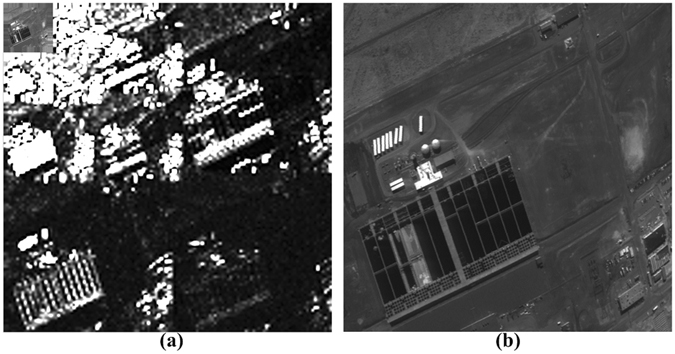
A saliency information map (**a**) and the corresponding reconstructed image (**b**).

In this paper, experimental tests of the proposed method based on remote sensing images are reported. A wavelet transform is applied to the remote sensing images to obtain BMPS images. Each image block in the BMPS images has dimensions of 16 × 16 pixels. Next, a DCT is applied to the BMPS images to obtain transformed post-wavelet coefficient blocks. After the DCT, an optimized saliency calculation method is applied to generate a saliency information map, as shown in Fig. 2(a).

**Figure 3:**
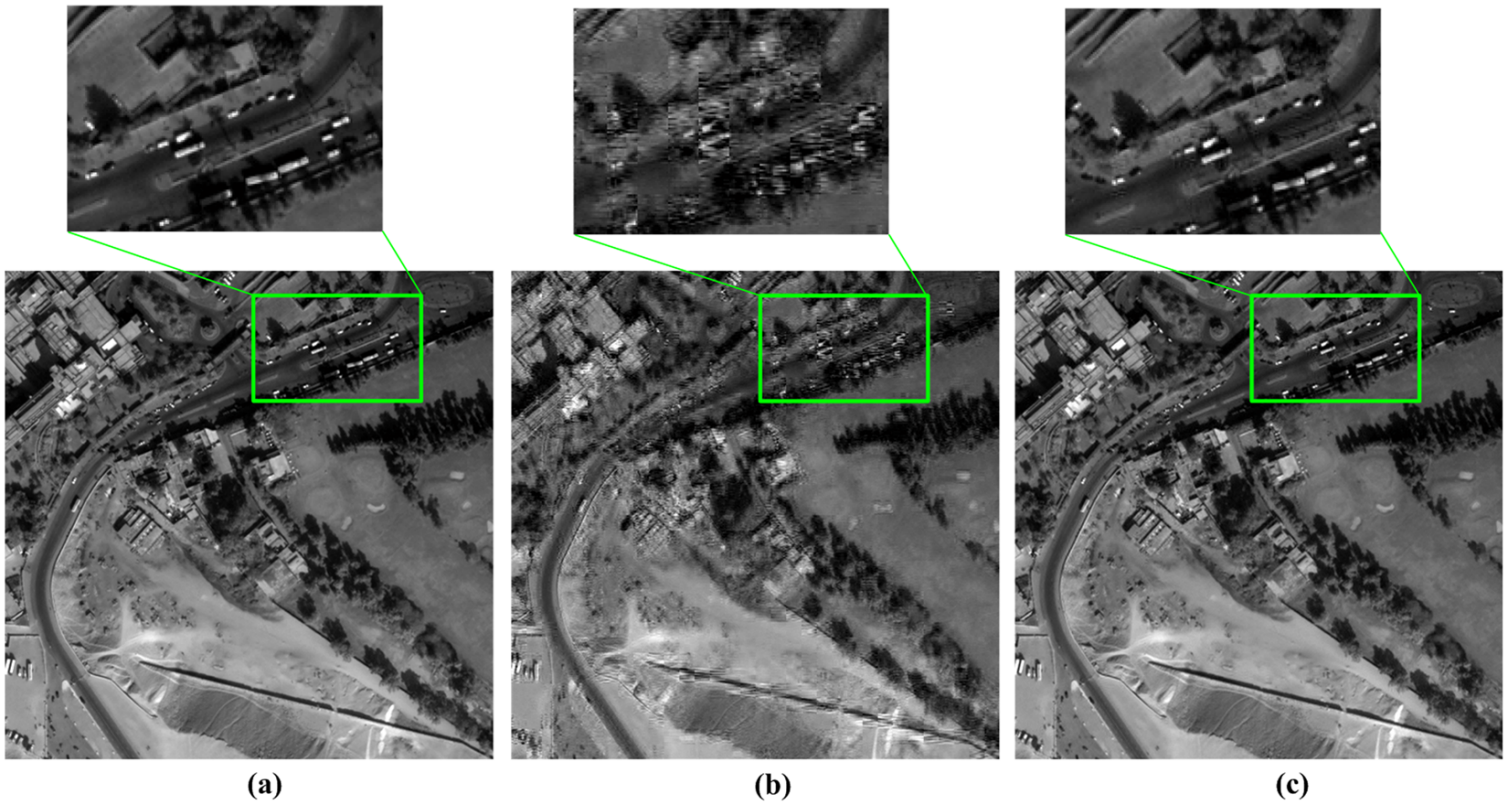
Reconstructed images obtained via PCT-CS and our method with M = 190 when the block size is 16 × 16 pixels: the original image (**a**); the reconstructed image obtained via PCT-CS, which exhibits blocking effects (**b**); and the reconstructed image obtained using our method, which does not exhibit blocking effects (**c**).

After the saliency information is calculated, a sensing measurement is performed on the transformed post-wavelet coefficient blocks to obtain sensing measurement images. Next, the sampled images are reconstructed using the orthogonal matching pursuit algorithm. The inverse DCT is computed to obtain the wavelet transform. Finally, the inverse DWT is computed to obtain the reconstructed image, as shown in Fig. 2(b). Figure 3 shows the reconstruction results of two saliency-based CS methods. As seen from the magnified regions of the three images, the reconstructed images obtained using the PCT-CS method exhibit blocking effects.

**Figure 4:**
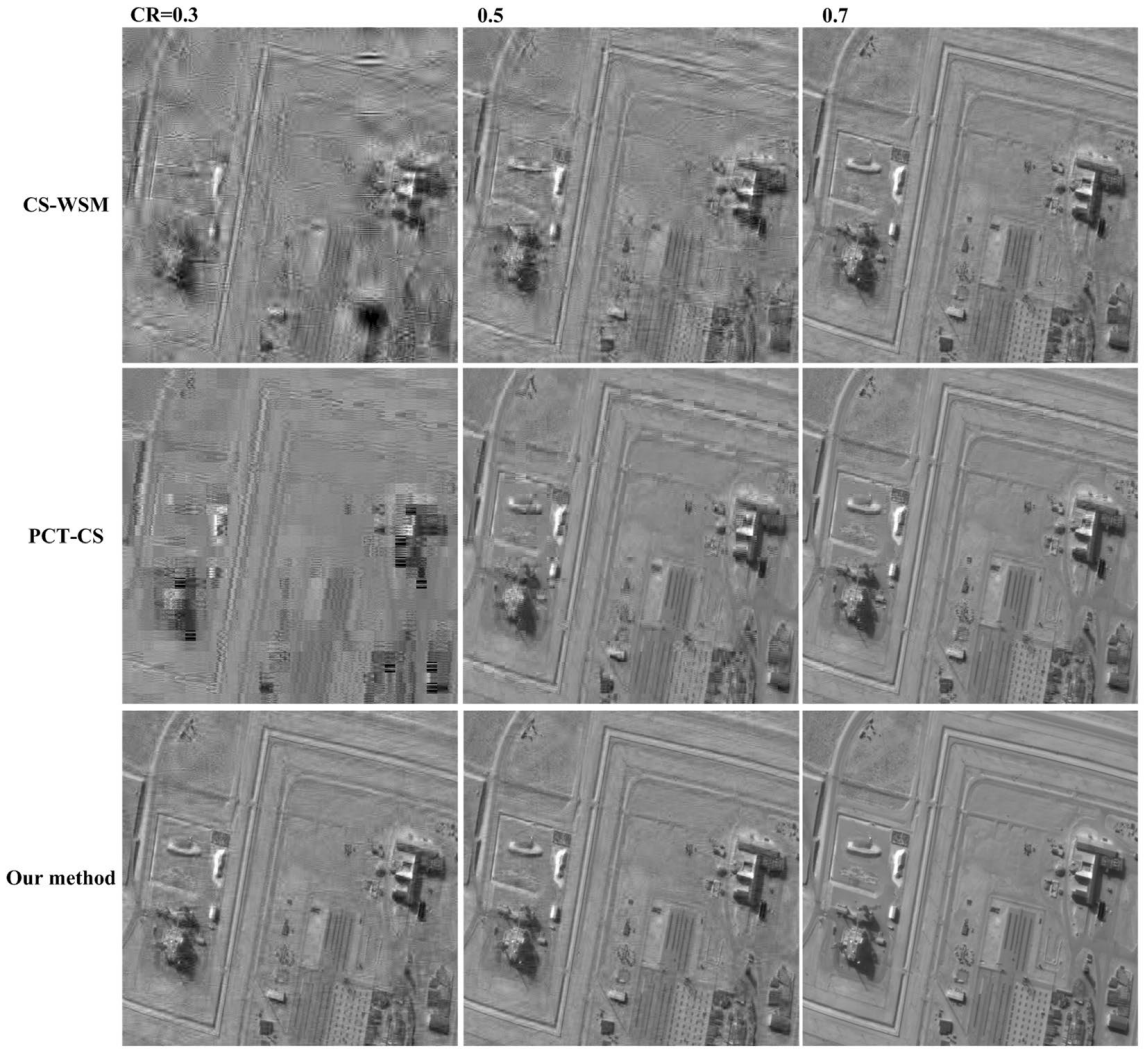
Reconstructed images obtained using different methods at different CR values.

To test the performance of our method (a new post-wavelet saliency algorithm), we compare our method with two other CS algorithms, namely, PCT-CS^9^ and CS without a saliency map (CS-WSM). These three methods were applied to generate multiple reconstructed images for statistical analysis. As the first step, the reconstructed images were analysed in terms of the peak signal-to-noise ratio (PSNR). The compression ratio (CR) is the ratio between the number of sensing measurements (samples) and the number of BMPS imaging units. The PSNR can be calculated as ${\rm{PSNR}}=10\cdot {\mathrm{log}}_{10}({{\rm{MAX}}}^{2}/{\rm{MSE}})$, where MAX is 2^*B*^−1, with *B* being the bit depth of each pixel, and MSE is the squared error between the original and reconstructed images. Because the bit depth of the test images used in this study was 8 bits, the PSNR was set to $10\times {\rm{1og}}({255}^{2}/{\rm{MSE}})$. A test image database of 150 remote sensing images depicting a variety of urban and natural scenes was used to measure the corresponding PSNRs. All images have dimensions of approximately 512 × 512 pixels. The average PSNR value was calculated as the final PSNR. The PSNRs calculated for the three different methods are shown in Table 1. Figure 4 Shows several images reconstructed using the different methods at different CRs.

**Table 1:** PSNRs of Three Different Methods.

CR	CS-WSM (*dB*)	PCT-CS (*dB*)	Our method (*dB*)
**0.30**	20.81	21.13	**28.45**
**0.40**	23.61	25.56	**29.96**
**0.50**	25.43	27.82	**31.32**
**0.60**	27.88	29.27	**33.09**
**0.70**	31,75	32.99	**34.87**
**0.80**	34.89	37.38	**38.42**

**Figure 5:**
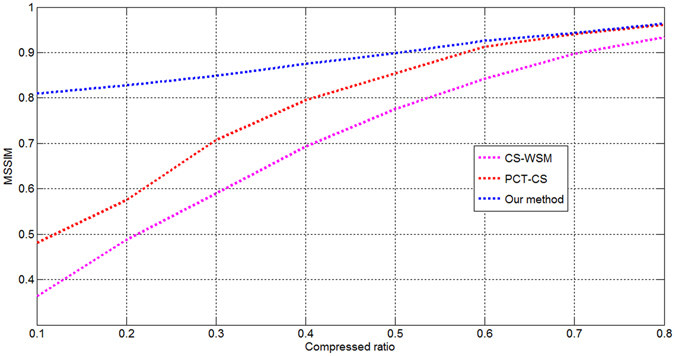
MSSIM values at different compression ratios.

**Figure 6:**
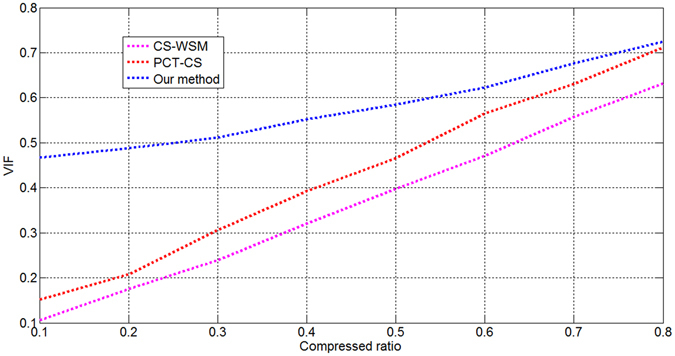
VIF values at different compression ratios.

Two additional image quality assessment indexes were also analysed: the mean structural similarity index (MSSIM)^13^ and the visual information fidelity (VIF)^14^. The MSSIM and VIF are defined based on the structural information extracted by the HVS. The MSSIM values at different compression ratios are shown in Fig. 5, and the VIF results are shown in Fig. 6. Finally, we tested and analysed our approach on a self-developed CS testing platform to verify its performance and feasibility. The testing platform includes image simulation sources, an S-CS system, a CS server, a decoding unit, and a display system. The CS server produces simulated images, which are transmitted to the image simulation source unit. The image simulation source unit adjusts the output line frequency, image size, and output time to simulate the output of a CCD sensor. The CS system uses a Virtex-PRO Xilinx FPGA with a 32-bit MicroBlaze as its processor. The simulated images are 3072 × 1024 pixels in size. The depth of each pixel is 8 bits per pixel. The compressed bitstreams are decoded by the decoding unit. The CS server injected remote sensing images with different texture information into the image simulation source unit to test the performance of the proposed approach. The proposed method offers the complete integration of imaging and compression, which is useful for improving the degree of integration of a micro-camera system. We used two transform-based coding methods, JPEG2000^15^ and CCSDS-IDC^16^, for a comparison of the compression performance. Table 2 shows the results obtained for the different algorithms.

**Table 2:** PSNRs of Three Different Methods.

Bit rate	CCSDS-IDC (*dB*)	JPEG2000 (*dB*)	Our method (*dB*)
**0.5**	25.98	27.42	**27.54**
**0.8**	29.70	30.36	**30.37**
**1.1**	32.58	34.47	**34.91**
**1.4**	34.16	36.64	**36.96**
**1.7**	35.55	37.37	**37.75**
**2.0**	36.78	38.17	**38.63**

To test the compression time of our method, the CCD line frequency was set to 7.2376 kHz. The size of each band was 3072 × 128 pixels. We compare the compression time of our algorithm with those of the other algorithms in Table 3. The processing time of our algorithm is 0.024 µs per sample, which is shorter than those of the other methods. Consequently, the compression of 3072 × 128 pixels requires only 8.25 milliseconds. In future applications, this algorithm could be optimized further on an FPGA on the basis of the CCD imaging principle.

**Table 3:** Compression Times of Different Algorithms.

Method	Compression time (*µs/sample*)
Our method	0.024
CCSDS-IDC^17^	0.025
JPEG2000^18^	0.11

## Discussion

As seen from Table 1, the results of PCT-CS are more satisfactory than those of CS-WSM. The experimental results of our method are the best among the three tested methods. The average PSNR values for our method are higher than those for the other two because our method possesses several advantages compared with the others. First, the saliency calculation method is more efficient, meaning that more high-frequency information is retained in the reconstructed images. Second, the reconstructed images do not exhibit blocking effects because the DCT and CS processes are performed in the wavelet domain rather than in the original image domain. Third, any remaining redundancies between local neighbouring wavelet coefficients can be exploited because the wavelet coefficients are further transformed by the DCT and CS processes. Fourth, a sparser representation can be achieved in the CS process because it is performed in the post-wavelet domain. These advantages ensure that the proposed method can achieve a higher PSNR at a given CR. As seen from Figs 3~6, the method proposed in this paper uses the most optimal CS techniques, utilizing the post-wavelet method to perform CS and the transformed coefficient method for the saliency calculation; thus, compared with the traditional saliency-based approaches, our proposed method achieves better results. The results presented in Table 2 indicate that the proposed method performs slightly better than the JPEG2000 algorithm and yields improvements of between 0.66 dB and 2.48 dB compared with the CCSDS-IDC method. These results demonstrate that the edge and texture information is preserved in the reconstructed images.

According to the test results presented in Table 3, our method requires the shortest processing time because it utilizes only sensing measurements in the encoding process. For the decoding process, our method requires a relatively longer time because it uses an OMP-based algorithm to recover the original images. The complexity of the encoding part of our method is low, whereas the decoding part has a higher complexity. Conventional compression algorithms usually encode the transformed coefficients using bit-plane encoding^17^ or embedded block coding with optimization truncation^18^, and the complexities of the encoder and decoder are basically the same. In our method, some of the complexity of the encoder is transferred to the decoder, which is very suitable for in-orbit applications because the encoder usually operates on a satellite platform with limited resources (such as power, memory and processing capacity), whereas the decoding operations are performed on the ground, where processing resources are not limited and multiple cutting-edge technologies (such as graphical processing units (GPUs)) can be utilized to speed up the decoding process.

## Conclusions

In this paper, we propose the PTFS-CS method, which treats wavelet coefficients as the pixels of a block-wise megapixel sensor and utilizes the spatial frequency in the post-wavelet domain to calculate the saliency information to guide the allocation of more sensing resources to salient regions than to non-salient regions. We prove that, as demonstrated by our experimental results, the use of frequency saliency for the allocation of sensing resources in the post-wavelet domain for remote sensing cameras can yield better images in terms of the PSNR, MSSIM, and VIF. The proposed post-wavelet saliency algorithm outperforms CS-WSM and PCT-CS and can satisfy the high quality requirements of a CS imaging camera. Thus, the proposed method offers significant benefits for future research based on remote sensing image analysis.

## Method

### S-CS based on a post-wavelet transform

**Figure 7:**
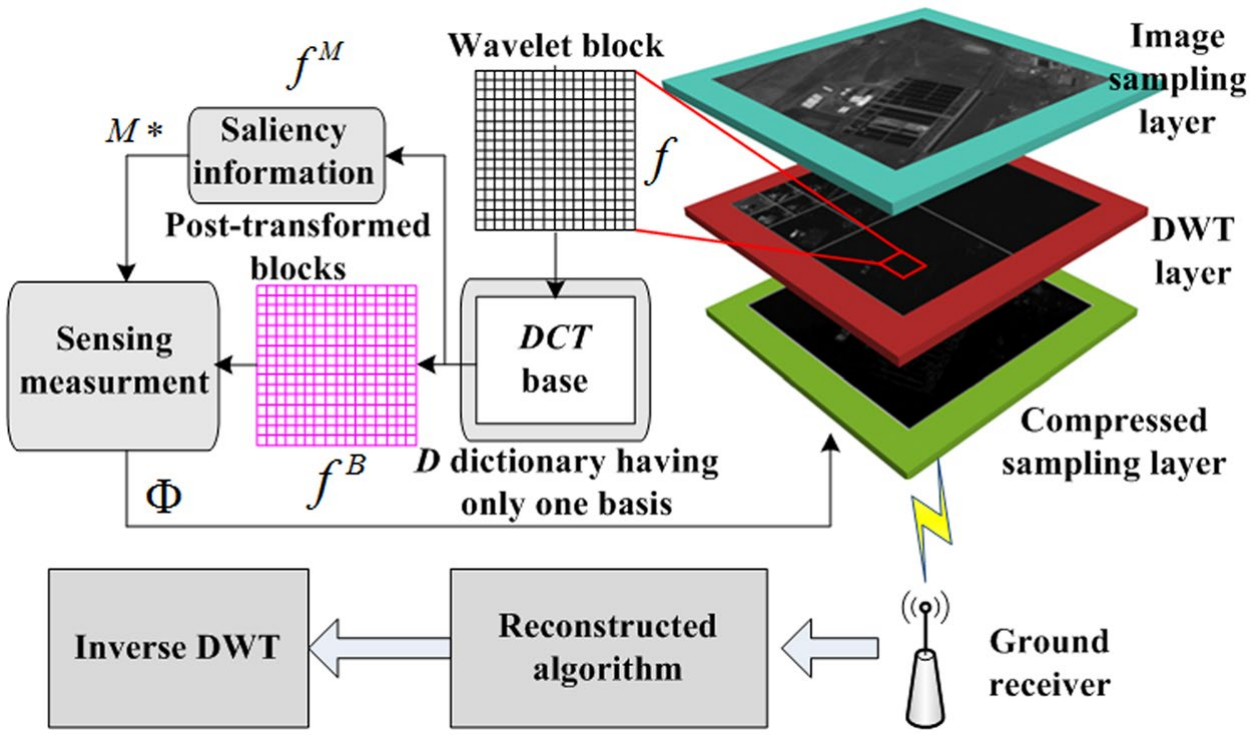
Principle of S-CS based on post-wavelet processing.

Figure 7 illustrates the principle of S-CS based on a post-wavelet transform. The BMPS consists of three layers, namely, an image sampling layer, a discrete wavelet transform (DWT) layer, and a CS layer. Photoelectric conversion and an analogue-to-digital (AD) converter are applied in the image sampling layer to digitize the image data; the digitized image data are regarded as the original images. In the DWT layer, a DWT is applied to the original images to extract wavelet sub-bands. In this paper, these wavelet sub-bands are defined as the BMPS images. Next, a DCT is applied to the BMPS images to obtain the DCT coefficients. The DCT is performed in the DWT domain. Therefore, we regard the DCT coefficients as the post-wavelet transformed coefficients. In our method, CS is performed in the post-wavelet transform domain based on the saliency information, as shown in Fig. 7.

In the post-wavelet S-CS process, each image block in the BMPS images contains 4 × 4 wavelet coefficients, and it is further transformed by using a basis function to perform S-CS. Theoretically, this basis function can be chosen to be a DCT, a bandelet basis function, principal component analysis, or a Hadamard transform. In this paper, we use the DCT as the basis function. For the implementation of post-wavelet S-CS, the different imaging units of the BMPS are denoted by LH_*i*_, HL_*i*_, and HH_*i*_, *i* = 1, 2, 3. In this paper, each imaging unit is divided into several blocks, denoted by *I*, with *N*_*I*_ coefficients (specifically, *N*_*I*_ = 256). To simplify the calculation, in each imaging unit of the BMPS, the *N*_*I*_ coefficients are divided into 16 coefficient blocks, which are called “wavelet blocks”. As *N*_*I*_ is set to 256 in this paper, each wavelet block is composed of 4 × 4 wavelet coefficients. When the distance (difference in coefficients) between adjacent wavelet blocks is greater than four pixels, the coefficient correlation between the adjacent wavelet blocks is relatively weak. Let *f* represent one wavelet-transformed block, which includes 16 wavelet coefficients that are expressed as *N*(*N* = 16) vector elements. Therefore, *f* can also be considered as a vector with *N* elements in R^N^. Let *D* be the post-wavelet transform dictionary, and let *B* denote the orthonormal basis function. The orthonormal basis consists of *N* basis vectors. Let the orthonormal basis be *φ*_*n*_, $n\in [1,N]$. Then, $={\{{\phi }_{n}\}}_{n=1}^{N}$. Let *f* ^*b*^ be a post-wavelet transformed coefficient block. Then, the post-wavelet transform can be expressed as

2 $${f}^{b}={\sum }_{n=1}^{16}\langle f,{\varphi }_{n}^{b}\rangle \cdot {\varphi }_{n}^{b}.$$

In each imaging unit of the BMPS, the post-wavelet transform is applied to all wavelet blocks. Let *F* be the set of transformed post-wavelet blocks from one imaging unit of the BMPS. Then, $F=\{{f}_{1}^{b},{f}_{2}^{b},\cdots ,{f}_{{N}_{b}}^{b}\}$, where ${N}_{b}({N}_{b}=16)$ is the number of transformed post-wavelet blocks in *F*. Let *Ω* be the saliency map of the BMPS. Therefore, $\Omega ={\{{F}_{q}\}}_{q=1}^{Q}$, where *Q* is the number of imaging units in the BMPS. Let $\Gamma =\{{\tau }_{1},{\tau }_{2},\cdots ,{\tau }_{{M}_{q}}\}$ represent a sensing measurement matrix, where *M*_*q*_ is the number of sensing measurements and satisfies *M*_*q*_ ≪ *N*_*I*_. *M*_*q*_ is determined by the saliency map, and ${M}_{q}\propto {F}_{q}$. Thus, the CS process can be expressed as $Y=\Phi {f}^{b}=\Phi \Psi f=\Phi ^{\prime} f$, where *Y* is the new sample, *Φ* is the sensing basis function, Ψ is the representation basis function, and *Φ*′ is an incoherent sampling matrix. Because *M*_*q*_ ≪ *N*_*I*_, image compression is achieved. After the CS process, the new sample *Y* is used to recover the original signal. Generally, the recovery of a signal $\tilde{{f}^{b}}$ from *Y* may be an ill-posed problem because *M*_*q*_ ≪ *N*_*I*_. However, such recovery is possible when the sensing basis function *Φ* and the representation basis function *Ψ* are incoherent, i.e., when any two vectors of *Φ* and *Ψ* are weakly correlated. With high probability, the incoherent sampling matrix *Φ′* possesses the restricted isometry property (RIP) when ${M}_{q}\gg c\times K\times \,\mathrm{log}({N}_{I}/K)$, where *c* is a positive constant and *K* is the sparsity coefficient^11, 12^. If the RIP is satisfied, then an accurate reconstruction signal $\tilde{{f}^{b}}$ can be reconstructed via $\tilde{{f}^{b}}=\Psi \,\ast \,\tilde{x}$, where $\tilde{x}$ is the solution to the *l*_1_ norm minimization problem, $\tilde{x}=\text{arg}{}_{x^{\prime} }{}^{{\rm{\min }}}\Vert x^{\prime} \Vert _{{l}_{1}}$, *s.t*. $y=\Phi \text{'}x\text{'}$.

### Fast saliency algorithm using spatial frequency

The saliency information is calculated in the post-wavelet transform domain via CS in the BMPS channel instead of the LR sensor channel. We use *f*′ to represent the two-dimensional BMPS image signal. The post-transformed coefficient matrix *g* can be obtained from $\psi f^{\prime} \psi$ ($g=\psi f^{\prime} {\psi }^{T}$), where *ψ* is the basis matrix in *D* (the post-wavelet transform dictionary). Because the basis matrix in *D* is the DCT basis, *ψ* is an orthogonal transform matrix of the DCT that consists of the cosine coefficients, and *ψ* ^*T*^ consists of the transposed coefficients. As the basis matrix is orthogonal, the inverse matrix of *ψ* is equal to its transpose $({\psi }^{-1}={\psi }^{{\rm{T}}})$. Consequently, the relationship between *f*′ and *g* can also be expressed as $g={\psi }^{T}f^{\prime} \psi$. In the above-described transformation process, the total energy of the image *f*′ is constant before and after the transformation. An important energy constraint between *f*′ and *g* can thus be built as follows:

3 $${\sum }_{i}{\sum }_{j}f^{\prime} {(i,j)}^{2}={\sum }_{i}{\sum }_{j}g{(i,j)}^{2}.$$

This energy constraint can also be expressed as $\text{trace}(f^{\prime} f{^{\prime} }^{{\rm{T}}})=\text{trace}(g{g}^{{\rm{T}}})$, where trace(·) represents the trace operation for a matrix. The derivative or gradient is a natural basis for the spatial features of an image. The gradient of an image can be used to calculate its spatial frequency, which reflects the saliency of the image. In this paper, we use the frequency saliency information of each BMPS block, which can be expressed as

4 $${E}^{2}={E}_{H}^{2}+{E}_{V}^{2}=\frac{1}{I\times J}\times (\sum _{x=2}^{I}\sum _{y=1}^{J}{|\partial {f}_{x}|}^{2}+\sum _{x=1}^{I}\sum _{y=2}^{J}{|\partial {f}_{y}|}^{2}),$$

where *E*_*H*_ and *E*_*V*_ are the horizontal and vertical frequencies, respectively, of the image block; $\partial {f}_{x}=f^{\prime} (x,y)-f^{\prime} (x-1,y)$; and $\partial {f}_{y}=f^{\prime} (x,y)-f^{\prime} (x,y-1)$. In each block, we let ${\rm{\Delta }}{f}_{x}$ and ${\rm{\Delta }}{f}_{y}$ denote the difference matrices in the horizontal and vertical directions, which are composed of the $\partial {f}_{x}$ and $\partial {f}_{y}$ values, respectively. The cyclic matrix *K* is expressed as

5 $$K={[\begin{array}{ccccc}-1 & 0 & \mathrm{..}. & 0 & 0\\ 1 & -1 & \mathrm{..}. & 0 & 0\\ 0 & 1 & \mathrm{..}. & 0 & 0\\ 0 & 0 & \mathrm{..}. & -1 & 0\\ 0 & 0 & \mathrm{..}. & 1 & 0\end{array}]}_{I\times J}.$$

On the basis of (5), the saliency components can be calculated as ${\rm{\Delta }}{f}_{x}=f^{\prime} K$ and ${\rm{\Delta }}{f}_{y}={K}^{T}f^{\prime}$. Thus, the following two saliency equations can be obtained:

6 $${\rm{\Delta }}{f}_{x}=f^{\prime} K={\psi }^{T}g\psi {\psi }^{T}P\psi ={\psi }^{T}gP\psi ,$$

7 $${\rm{\Delta }}{f}_{y}={K}^{T}f^{\prime} ={({\psi }^{T}P\psi )}^{T}{\psi }^{T}g\psi ={\psi }^{T}{P}^{T}g\psi ,$$

where *P* is the transformed coefficient matrix of *K*. After the application of a $4\times 4$ DCT, *P* is expressed as the following matrix: ${P}_{4\times 4}=[0\,0\,0\,0;-0.6533-0.2929\,0.2706\,0;0-0.9239-1\,0.3827;-$ ${0.27060-0.6533-1.7071]}_{4\times 4}$ *P*.As described in (6) and (7), *gP* and ^*T*^ *g* are the transformed coefficients of ${\rm{\Delta }}{f}_{x}$ and ${\rm{\Delta }}{f}_{y}$, respectively. Therefore, (4) can be expressed as

8 $$\begin{array}{rcl}\frac{1}{I\times J}\times \sum _{x=1}^{I}\sum _{y=1}^{J}{|\partial {f}_{x}|}^{2} & = & \frac{1}{I\times J}(trace(\nabla {f}_{x}{(\nabla {f}_{x})}^{T}))\\  & = & \frac{1}{I\times J}(trace[gP{(gP)}^{T}])=\frac{1}{I\times J}[trace(gP{P}^{T}{g}^{T})],\end{array}$$

9 $$\begin{array}{rcl}\frac{1}{I\times J}\times \sum _{x=1}^{I}\sum _{y=1}^{J}{|\partial {f}_{y}|}^{2} & = & \frac{1}{I\times J}(trace({(\nabla {f}_{y})}^{T}\nabla {f}_{y}))\\  & = & \frac{1}{I\times J}(trace[{({P}^{T}g)}^{T}{P}^{T}g])=\frac{1}{I\times J}[trace({g}^{T}P{P}^{T}g)].\end{array}$$

The frequency saliency information can also be expressed as $\frac{1}{I\times J}[trace(gP{P}^{T}{g}^{T})+trace({g}^{T}P{P}^{T}g)]$. Let $Q=P{P}^{{\rm{T}}}$, where the matrix *Q* is constant because *P* is the transformed coefficient matrix of *K*. Next, *K*can be transformed by using an 8 × 8 or 4 × 4 DCT to obtain the fixed matrix shown below: ${Q}_{4\times 4}={[0000;00.585800;0020;0003.4142]}_{4\times 4}$. In (9), the matrix *Q* is a diagonal matrix. Thus, (4) can be expressed as

10 $$\begin{array}{rcl}{E}^{2} & = & \frac{1}{I\times J}[trace(gQ{g}^{T})+trace({g}^{T}Qg)]=\frac{1}{I\times J}[trace(Qg{g}^{T})+trace(Q{g}^{T}g)]\\  & = & \frac{1}{I\times J}[\sum _{u=1}^{M}\sum _{v=1}^{N}Q(u,u)\times g{(u,v)}^{2}+\sum _{u=1}^{M}\sum _{v=1}^{N}Q(v,v)\times g{(u,v)}^{2}]\\  & = & \frac{1}{I\times J}[\sum _{u=1}^{M}\sum _{v=1}^{N}R(u,v)\times g{(u,v)}^{2}],\end{array}$$

with

11 $${R}_{4\times 4}={[\begin{array}{cccc}0 & 0.5858 & 2.0000 & 3.4142\\ 0.5858 & 1.1716 & 2.5858 & 4.0000\\ 2.0000 & 2.5858 & 4.0000 & 5.4142\\ 3.4142 & 4.0000 & 5.4142 & 6.8284\end{array}]}_{4\times 4}.$$

According to (10), the saliency information in the frequency domain for each BMPS block can be expressed in terms of its transformed coefficients. Moreover, the relationship between the bit rate *r* and the number *M* of nonzero post-transformed coefficients quantized using a nearly uniform step quantization can be expressed as *r* ≈ *γ*_0_ *M*, where *γ*_0_ is a constant parameter. The post-transformed coefficients *g*(*u*, *v*) in (10) can be replaced with

12 $${I}_{m}=\{\begin{array}{c}1,\lfloor |g(u,v)|\rfloor \ne 0\\ 0,\lfloor |g(u,v)|\rfloor =0\end{array}.$$

According to (12), the elements of *g*(*u*, *v*) only take values of “1” and “0”; thus, the saliency information can be rapidly calculated, and the calculation can be easily implemented in hardware systems. After the saliency map is established based on Eqs (10) and (12), the sensing resources are allocated according to the established saliency map. Each imaging unit of the BMPS consists of a 16 × 16 pixel block. Considering that the post-transform saliency CS process is performed in the post-wavelet domain, the smallest number of BMPS sensing measurements (samples) is allocated to the lowest-resolution post-wavelet sub-band. For the post-wavelet sub-bands at other resolutions, the CS matrices have different sizes because the different imaging units contain different amounts of information. The sensing sampling process can be expressed as ${[{Y}_{1},{Y}_{2},\ldots ,{Y}_{{\rm{L}}}]}^{{\rm{T}}}={\rm{diag}}({\Phi }_{1},{\Phi }_{2},\ldots ,{\Phi }_{{\rm{L}}}){[{X}_{1},{X}_{2},\ldots ,{X}_{L}]}^{{\rm{T}}}$, where *L* is the number of imaging units of the BMPS, diag(·) denotes a diagonal matrix, and *Φ*_*P*_ (*p* = 1, 2, …, *L*) is the CS sensing matrix of the *p*th imaging unit and is determined based on the saliency information map. Finally, the measured data sample *Y* is processed via quantization, entropy coding and packing to obtain the final encoded bitstream, which can be reconstructed by a recovery algorithm on the ground.
